# Late Triassic *†Cryptovaranoides microlanius* is a squamate, not an archosauromorph

**DOI:** 10.1098/rsos.231874

**Published:** 2024-11-27

**Authors:** David I. Whiteside, Sofía A. V. Chambi-Trowell, Michael J. Benton

**Affiliations:** ^1^ School of Earth Sciences, University of Bristol, Bristol BS8 1RJ, UK; ^2^ Fossil Reptiles, Amphibians and Birds Section, The Natural History Museum, Cromwell Road, London SW7 5BD, UK

**Keywords:** phylogenetics, divergence times, Squamata, reptiles, Triassic, *†Cryptovaranoides*

## Abstract

*†Cryptovaranoides microlanius* from the latest Triassic of England was described in 2022 as a crown-clade squamate, of importance as the oldest such modern-type lizard, extending their temporal range downward by 35 Myr. This view was challenged in 2023, and *†Cryptovaranoides* was reinterpreted as an archosauromorph. These decisions matter because the original view has an impact on our understanding of the early stages of squamate evolution; the revised view removes the species from such a role. The revisers emphasized the need to make careful observations of the fossils and to interpret the morphological data appropriately in terms of relationships; here, we find many errors of observation and interpretation in the work of the revisers, and we correct these with reference to the fossils, both in the rock and in the computed tomography scans we had made for the original description. We show that when the observational errors are corrected and the taxa recoded, every phylogenetic analysis confirms our original conclusion that *†Cryptovaranoides* is not an archosauromorph, but a lepidosauromorph, a lepidosaur, a pan-squamate and a crown squamate.

## 1. Introduction

We presented a detailed description of a new, small diapsid reptile from the latest Triassic (202 Ma) of South Gloucestershire, near Bristol, southwest England, providing evidence that not only was this a new genus and species (*†Cryptovaranoides microlanius*) but it was also a pan-squamate and indeed a crown squamate, with affinities to the Anguimorpha [1]. We defined our [1] terminology using recent definitions of the clades and apomorphies from the *Phylonyms* book [2–4]. Squamata is the maximum crown-clade and Pan-Squamata includes Squamata and its stem group, as well as all Squamata and extinct species that are more closely related to the crown-clade than to *Sphenodon punctatus*. The importance of the find was that it shifted the origin of Squamata back by 35 Myr from the formerly oldest fossils in the Middle Jurassic, and so it reset the timing of origins of several major clades within Squamata to much more ancient dates than had been understood previously. In a response [5], the argument is made that *†Cryptovaranoides* is not a squamate, and not even a lepidosauromorph, but instead is an archosauromorph. If supported, this revision would imply profound errors in our original paper and removes the evidence for a Late Triassic crown squamate. On the other hand, many stem pan-squamates had been reported already from the Triassic [6–10] and the origin of Pan-Squamata was at least in the Middle Triassic (>240 Ma) based on an early rhynchocephalian fossil [6].

The new work [5] proposes substantial corrections to our earlier work [1], but we find that much of the critique is not as strong as suggested, and much is incorrect. Therefore, we have arranged this response in order of their most conspicuous errors first, followed by features where the evidence is clearly against their views. We finish with equivocal characters, where the fossil material is not good enough to be sure.

We also present our phylogenetic analysis where we score characters in a conservative manner, meaning that we include only those character states recorded from the holotype or isolated bones that are directly referable to holotype elements. Our conclusion is that none of the new evidence [5] on the affinities of *†Cryptovaranoides microlanius* opposes its assignment to crown-clade Squamata, as originally proposed [1]. We finally test the alternative views against established apomorphies of the key clades.

Institutional abbreviations: NHMUK, the Natural History Museum, London. PIMUZ, The Palaeontological Institute of the University of Zurich.

## 2. Major errors of observation by Brownstein *et al*. (2023)

### 2.1. Entepicondylar and ectepicondylar foramen of humerus

**Figure 1.**
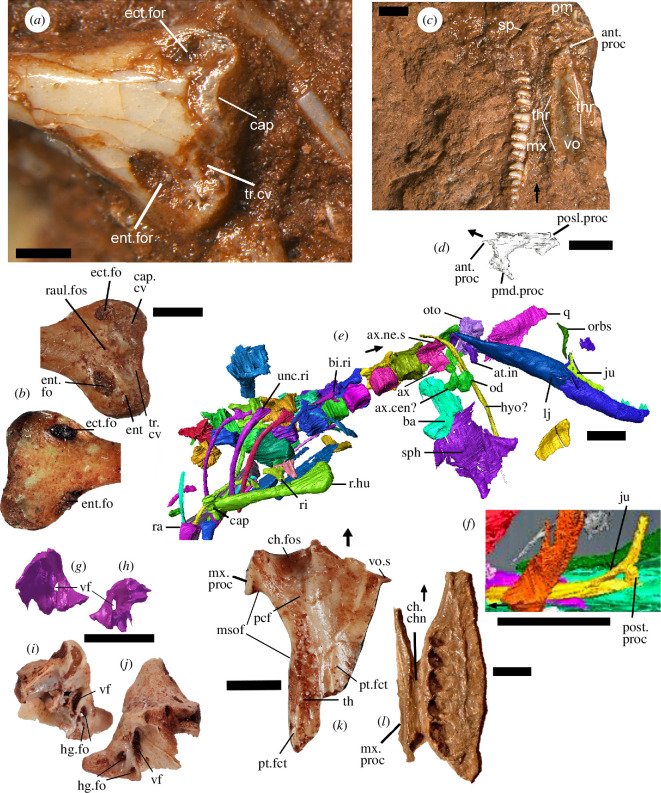
Images from the holotype NHMUK PV R36822 of *C. microlanius,* separate isolated bones referable to the taxon and a palatine of *†Tanystropheus longobardicus for* comparison. (*a*) Distal end of left humerus in anterior view exposed on the surface of the holotype rock showing an ectepicondylar and an entepicondylar foramen and the capitellum. (*b*) NHMUK PV 38911 isolated larger specimen of the distal end of left humerus of *†Cryptovaranoides microlanius* in (above) anterior and (below) posterior views showing similar features except the condyle of the capitellum. (*c*) Right maxilla, right vomer in ventral view, right septomaxilla in dorsal view and both premaxillae exposed on the surface of the holotype rock. (*d*) Scan of right septomaxilla in posteromedial view. (*e*) Scan of NHMUK PV R36822, mainly in ventral view, showing right lower jaw, orbitosphenoid, right jugal, right quadrate, basioccipital, sphenoid, anterior vertebrae and ribs as well as pectoral and forelimb bones. (*f*) Scan of left jugal in lateral view for comparison. (*g,h*) Fragment of right otoccipital from scan of NHMUK PV R36822 showing position of vagus foramen, in (*g*) medial and (*h*) lateral views. (*i,j*) NHMUK PV 38889, fragment of left exoccipital part of otoccipital in (*i*) lateral and (*j*) medial views, annotated to show vagus foramen. (*k*) Isolated right palatine NHMUK PV R 38916 of *†Cryptovaranoides microlanius* in ventral view, showing extent of the choanal sulcus (fossa). (*l*) Right palatine of *†Tanystropheus longobardicus* PIMUZ T 2484 showing position of choanal channel. Scale bars all 2 mm except (*a*) = 0.5 mm and (*d*) = 1 mm. Arrows show anterior.ant, anterior; at, atlas; ax, axis; ba, basioccipital; bi, bicapitate; cap, capitellum; cen, centrum; ch, choana; chn, channel; cv, cavity; ect, ectepicondyle; ent, entepecondyle (entepicondylar); fct, facet; fo, foramen (foramina); for, foramen; fos, fossa; hg, hypoglossal; hu, humerus (humeral); hyo, hyoid; in, intercentrum; ju, jugal; l., left; lj, lower jaw; msof, margin of suborbital fenestra; mx, maxilla (maxillary); ne, neural; od, odontoid; orbs, orbitosphenoid; oto, otoccipital; pcf, posterior of the choanal fossa; pm, premaxilla; posl, posterolateral; post, posterior; pmd, posteromedial; proc, process; pt, pterygoid; q, quadrate; r, right; ra, radius; raul, radioulnar; ri, rib; s, spine; sp, septomaxilla; sph, sphenoid; th, tooth (teeth); thr, tooth row; tr, trochlea; unc, unicapitate; vf, vagus foramen; vo, vomer (vomerine).

These structures ([7], ch. 309; [10], ch. e305, 307; [5], ch. 308, 310), often regarded as key lepidosaur and squamate characteristics (although the entepicondylar foramen is lost in squamates [11]), are in fact present in *†Cryptovaranoides*. Brownstein *et al.* [5] state that ‘their inspections of the CT scans did not reveal any detectable foramina on the entepicondyles or ectepicondyles’ or remarkably they ‘were unable to observe any foramina in Whiteside *et al.* (2022) figures’. In fact, despite sediment occupying the deeper parts of the passages, both foramina are clearly visible on the left humerus of the holotype [1, fig. 1A,D]. The foramina are obvious, especially in the holotype left humerus when the specimen is viewed directly (figure 1*a*) and Brownstein *et al*. [5] were able to obtain photographic images of the holotype specimen before acceptance of their paper. Furthermore, we figure a much larger separate left humerus NHMUK PV R38911 (figure 1*b*) preserved in excellent detail which shows the foramina openings on both the anterior and posterior distal surfaces. Another, acid prepared specimen NHMUK R38929 has the forame cleared of sediment and it forms a continuous entepicondylar passage. The presence of entepicondylar and ectepicondylar foramina in *†Cryptovaranoides microlanius* is undeniable and pronounced.

### 2.2. Septomaxilla

Similarly, the claim that the septomaxilla ([10], ch. 10−13; [5], ch. 9−12) is absent [5] is simply wrong. Indeed, they fail to show the septomaxilla in their images [5, fig. 1a–c], but we labelled it as a slightly disarticulated element in proximity with, and between, the premaxilla and maxilla [1, fig. 1B]. It is a typical septomaxilla (figure 1*c*,*d*), triangular, arrow-shaped with anterior, posterolateral and posteromedial processes; it is also curved dorsally. We further illustrate the position and morphology of the septomaxilla in the holotype (figure 1*c*,*d*). Additional evidence comes from an isolated maxilla of a larger *†Cryptovaranoides* individual (NHMUK PV R36999; [1, fig. 3C]), which shows a septomaxillary facet. If Brownstein *et al.* [5] observed the septomaxilla in the holotype they made no mention of what they considered it to be if not a septomaxilla.

### 2.3. Expanded radial condyle of the humerus

We [1] noted an expanded radial condyle on the humerus ([10], ch. 308; [5], ch. 311), but Brownstein *et al*. [5] concluded this key squamate character is absent. However, the holotype specimen shows a radial condyle (=capitellum) projecting above the neighbouring region of the distal anterior extremity (figure 1*a*). Despite the juvenility of the specimen and some damage to the capitellum, we considered that its prominence supports the scoring of the expanded radial condyle as present. This is reinforced by the larger humerus (figure 1*b*) which is missing the condyle, as is typical in the preservation of fissure lepidosaurs (e.g. *†Clevosaurus*; [12, fig. 29b]) but the cavity in which it sat clearly indicates a substantial condyle in life. There is also a pronounced contouring with raised regions of the distal end of the right humerus shown in the scan (figure 1*e*) which are condyle structures, and we suggest includes the radial condyle.

### 2.4. Absence of jugal posterior process

Except for a very few fossil taxa, including two polyglyphanodontians, *†Tianyusaurus* and *†Polyglyphanodon* [13], squamates lack a posterior process on the jugal ([10], ch. 36, 37; [5], ch. 42−45). Brownstein *et al*. [5] suggested such a process might be present, stating that ‘the posterior region of the (left) jugal is broken on the holotype of *C. microlanius’* and that ‘it is possible that a posteroventral process was present but not preserved’. There is no evidence of a broken posterior process in their figures [5, fig. 1a,b] and they do not mention that both jugals are preserved in the holotype. The right jugal [1, fig. 1F] shows the same morphology as the left jugal (compare figure 1*e* with figure 1*f*), and it is preserved next to the right lower jaw and is protected by that bone and sediment. As neither jugal shows a posterior process (the posterior margin abruptly terminates) and are of near identical shape, we are confident that there is no missing posterior process. Loss of the posterior process of the jugal is a feature of Lepidosauria or Squamata, and is reversed in Rhynchocephalia, which have re-acquired the long posterior process of the jugal [14] and is an apomorphy of the clade. Simões *et al*. [7, ch. 36] considered only the overall presence or absence of the jugal posteroventral process, whereas we also code [10, ch. 369] for a complete or incomplete lower temporal bar. *†Cryptovaranoides*, on this basis, is placed within the Pan-Squamata [3] rather than its sister, the Rhynchocephalia.

### 2.5. Anterior emargination of the maxillary nasal process

This feature [10, ch. 19] is said to be ‘rarely observed in squamates but is a hallmark feature of archosauromorphs, where it contributes to the formation of the antorbital fenestra (or fossa when the fenestra is absent …’ [5]. There is no further description, and we cannot see this feature. Brownstein *et al*. [5] seemingly relied on the anteriorly broken left maxilla [5, fig. 1b] that lacks the anteriormost region to make this observation, apparently ignoring the more complete right maxilla (cf. both maxillae in [1, fig. 1b]). The anterior margin of the maxillary nasal process of the right maxilla tapers anteriorly [1, fig. 1b] with no evidence of the type of emargination suggested and would have connected to the nasal bone without a gap. The suggestion that this feature might imply an incipient antorbital fenestra or fossa is not explained and is counter to the evidence of the holotype.

## 3. Observations that suggest *†Cryptovaranoides* is a squamate

### 3.1. Subdivision of the metotic fissure

We next consider Brownstein *et al.*’s [5] comment that subdivision of the metotic fissure by the crista tuberalis into vagus (jugular) foramen and recessus scala tympani ([10], ch. 153, 382; [5], ch. 157) should be treated as a missing character. We took the character [8, ch. 153] as the basis to ascertain whether *†Cryptovaranoides* had a squamate-type divided metotic fissure. That character emphasized that a crista tuberalis separates the lateral opening of the recessus scala tympani (lrst) from the vagus (or jugular) foramen but only required the crista tuberalis to be present to be scored as 1. We recognized the lrst and crista tuberalis in *†Cryptovaranoides* and after considerable scrutiny of the broken region of braincase concluded that a vagus foramen was almost certainly present (although missing in NHMUK PV R37377). We were sure that was the case as we had a scan of the otooccipital fragment (figure 1*g*,*h*) next to the right quadrate of the holotype NHMUK PV R36822 [1, fig. 1C] which showed a recognizable vagus foramen. Although we did not give a detailed figure or description of that fragment [1], we were aware of this feature and that the metotic fissure was divided into vagus (jugular) foramen and recessus scala tympani. However, the resolution of the scan is not as good as we would have liked, so we searched the collection for an otoocipital fragment which was unequivocal in showing the vagus foramen (specimen NHMUK PV 38889; figure 1*i*,*j*). This new image confirms our original conclusion.

### 3.2. Fusion of exoccipitals and opisthotics forming an otoccipital

Brownstein *et al.* [5] do not query our recognition of this feature [10, ch. 151] but rather refer to its status as variable in squamates and in other reptiles, but they do not say which other reptiles. We are aware that fused exoccipitals and opisthotics are variable across Squamata, but it is a typical feature of the clade, as indicated also by de Queiroz & Gauthier [4] as one of the ‘more obvious diagnostic apomorphies of the Squamata’. Therefore, we are content to accept this character as indicative of Squamata.

### 3.3. Enclosed vidian canal exiting anteriorly at base of each basipterygoid process

It is hard to follow the enclosed vidian canal ([10], ch. 135; [5], ch. 139) because the holotype sphenoid, although preserved in three dimensions, is repeatedly cracked and sediment has deeply penetrated the interstices [1, fig. 5G–I]. Our position is, therefore, *contra* [5], who regard the sphenoid as ‘well-preserved in three dimensions’, and any discontinuous lacunae are ‘unlikely to be an artefact of crushing’. We annotated the anterior opening of the enclosed vidian canal from the scan images [1, fig. 5E,F] and supported this suggestion with the presence of enclosed foramina [1, fig. 5H,I] in that vicinity. Brownstein *et al*. [5] suggested that these anterior openings might be blind recesses but without further explanation. We further suggested that the posterior opening of the vidian canal may be at the position of the sphenoid wings or more anteriorly just posterior to the basipterygoid processes [1, fig. 5C,G]. The discovery of a very abraded but larger isolated sphenoid NHMUK PV R 37603a suggests both may well be correct as we recognize the track of the vidian canal from the wings before entering foramina at the posterior bases of the basipterygoid processes. The isolated sphenoid was scanned in the U.K. Diamond Light Source synchrotron facility at Harwell, but the bone is ultra-thin owing to abrasion during deposition, with large fragments missing and the dorsum sella has been obliterated so little additional evidence can be gathered. We consider that our original suggestion of an enclosed vidian canal exiting anteriorly at the base of the basipterygoid processes has sufficient supporting evidence to draw that conclusion. However, to be conservative we code the character as query (?) here.

### 3.4. Palatine choanal sulcus

In, their critique, Brownstein *et al*. [5] state that the palatine choanal sulcus (=fossa) ([10], ch. 100; [5], ch. 106) of *†Cryptovaranoides* is ‘anteroposteriorly restricted’ and that ‘it is mediolaterally restricted so that it barely fills half of the mediolateral length of the anterior margin of the palatine’. They argue that the latter condition is ‘rare or absent in living lizards’ but is found in ‘some archosauromorphs including *†Tanystropheus* and *†Macrocnemus’*. However, we note that it only measures 27−28% of the anterior width of the palatine in the living iguanid lizard *Ctenosaura* [15, fig. 4]. In our paper [1], we indicated that the holotype of *†Cryptovaranoides* is a juvenile where the choanal fossa is about 27% the anterior width of the palatine, very near that of a *Ctenosaura* adult.

However, Brownstein *et al.* missed our description [1] of larger unregistered bones in the collection in which ‘the posterior margin of the fossa extends posteriorly to the anterior edge of the suborbital fenestra/foramen’, as shown by the now registered NHMUK PV R 38916 in figure 1*k*. Here, the choanal sulcus is anteroposteriorly more extensive than in many typical living squamates (exceeding the proportional length in, e.g. *Iguana*, *Ctenosaura* and *Agama*) and moreover it constitutes about 31% of the mediolateral width of the anterior margin of the palatine. Therefore, adult *†Cryptovaranoides* shows a greater relative width of the choanal fossa relative to the mediolateral width of the anterior border of the palatine than in several well-known living lizards.

We confirm that the choanal sulcus is deep, as Brownstein *et al*. accept [5, p. 12], but we also suggest, *contra* [5], that it is as deep as in many living lizards (figure 1*k*). The sulcus in *†Cryptovaranoides* becomes less deep posteriorly as it does in modern lizards, and it does not remain as a fossa stretching right across the palatine anteroposteriorly; again, this is the case in extant squamates such as *Ctenosaura* [15]. Brownstein *et al*. [5] compare the *†Cryptovaranoides* palatine choanal fossae to those of the protorosaurs *†Tanystropheus* and *†Macrocnemus*, but do not provide details or illustrations. We suggest the comparison is spurious, as recent descriptions of the palatine of those taxa [16,17] show no choanal sulcus on the bone. We figure the ventral view of the right palatine of *†T. longobardicus* (figure 1*l*) which shows that the ‘choanal fossa’ is not a sulcus but is rather a channel running parallel to the maxillary process and, unlike in squamates, right across the palatine anteroposteriorly. Brownstein *et al*. [5, ch. 106] use the term ‘sulcus choanalis’, not channel, as the character, so describing the *†Tanystropheus* feature as a choanal sulcus is inappropriate. The *†Cryptovaranoides* palatine, and particularly the choanal sulcus (=fossa) is undoubtedly squamate in nature.

### 3.5. Vomer ventral ridges

We pointed out [1] that ventrally the vomer has three prominent posteroanterior longitudinal toothed ridges or crests (figure 1*c*) that converge somewhat anteriorly ([10], ch. 92, 93; [5], ch. 100), diagnostic of Anguimorpha within Squamata. Such structures might be present more widely among squamates [5]. However, we dispute the description of these vomers by Brownstein *et al*. [5] as flattened and like those of non-lepidosaurs. The *†Cryptovaranoides* vomer is highly contoured and has marked ventral ridges (and troughs) which are crested in both lateral and medial ridges, as seen better in the holotype specimen (figure 1*c*) than in the scans. Posteriorly, the middle of the three ridges broadens transversely like that of *Pseudopus apodus* [18, fig. 1B]. *Contra* Brownstein *et al*. [5], we describe the teeth as concentrated on the three ventral ridges which converge anteriorly (figure 1*c*); teeth of *P. apodus* are also ridge-based [18]. Brownstein *et al*. [5] regard the *†Cryptovaranoides* vomers as akin to those of *†Gephyrosaurus bridensis* but the vomer of the rhynchocephalian is flattened [19, fig. 22A,B] and the teeth are not concentrated on ridges. The vomer of *†Gephyrosaurus* also fundamentally differs from *†Cryptovaranoides* in the presence of a tube ([19], lateral cylinder) that forms its lateral dorsal margin, and this is absent in *†Cryptovaranoides* but is present in the sphenodontian rhynchocephalian *†Diphydontosaurus avonis* [14, fig. 20a].

### 3.6. Lacrimal arches dorsally over lacrimal duct and floors lacrimal duct with medial process posteriorly

**Figure 2.**
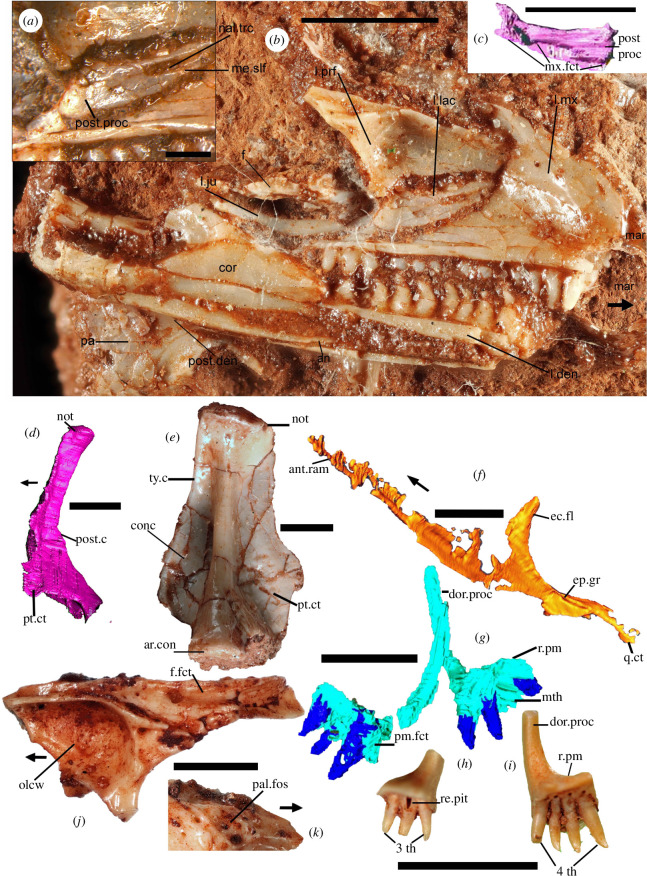
Photographs and CT scan images of NHMUK PV R36822 holotype bones of *†Cryptovaranoides microlanius* and isolated quadrate, prefrontal and premaxillae. (*a–c*) Left side of holotype skull and lower jaw of *†Cryptovaranoides microlanius*. (*a*) Close-up of left lacrimal in medial view. (*b*) Left side of skull and lower jaw in medial view. (*c*) Computed tomography (CT) scan of left lacrimal in lateral view. (*d*) Holotype right quadrate CT scan in medial view. (*e*) Isolated left quadrate NHMUK PV R 37606 digitally removed from matrix. (*f*) CT scan image of holotype right pterygoid in ventromedial view. (*g*) CT scan of holotype left and right premaxillae in posteroventral views. (*h*) Isolated left premaxilla NHMUK PV R 38914 in posteroventral view. (*i*) Isolated right premaxilla NHMUK PV R 38913 in posterior view. (*j*) Isolated right prefrontal NHMUK PV R 38912 in medial view. (*k*) Lateral view of right prefrontal NHMUK PV R 38912 showing palpebral fossa. Scale bars all 2 mm except (*a*) which is 0.5 mm. Arrows show anterior. an, angular; ant, anterior; ar, articular; c, crest; con, condyle; conc, conch; cor, coronoid; ct, contact; den, dentary; dor, dorsal; ec, ectopterygoid; ep, epipterygoid; f, frontal; fct, facet; fo, foramen (foramina); fos, fossa; gr, groove; ju, jugal; l., left; lac, lacrimal; mar, missing anterior region; me, medial; mth, missing tooth; mx, maxilla (maxillary); nal, nasolacrimal; not, notch; olcw, olfactory chamber wall; pa, palatine; pal, palbebral; pm, premaxilla; post, posterior; proc, process; prf, prefrontal; pt, pterygoid; ram, ramus; re, resorption; q, quadrate; r, right; slf, shelf, th, tooth (teeth); trc, tract; ty, tympanic.

Arguing that ‘this feature is unobservable and cannot be scored for this taxon, as this region of the skull is disarticulated, and the lacrimal region is fragmented’, Brownstein *et al*. [5, fig. 1] reference the scan images. However, the actual specimen shows the feature much more clearly; we show the left lacrimal (figure 2*a–c*) including detailed medial views (figure 2*a*,*b*) and the nasolacrimal tract is clearly walled posteriorly by the medial process of the lacrimal. *Contra* Brownstein *et al*. [5], we did not score this trait in any dataset, but rather it was observed as an anguimorph apomorphy when our phylogenetic analysis revealed the affinities of *†Cryptovaranoides* to the Anguimorpha.

### 3.7. Quadratojugal not present as a separate element

We coded the quadratojugal as absent ([10], ch. 38−42; [5], ch. 47−50) in *†Cryptovaranoides*, based on both the juvenile holotype specimen (figure 2*d*; [1, fig. 6E]) and in larger individuals (figure 2*e*). Disagreeing with our interpretation that the quadratojugal is not present as a separate element, Brownstein *et al*. [5] suggest that an ontogenetic sequence would be required to test this character, and on their logic, this would be a requirement for any fossil diapsid. Nonetheless, we argue, based on juvenile and adult specimens and the absence of a quadratojugal facet on the quadrate (figure 2*e*) that *†Cryptovaranoides* lacked a quadratojugal as a separate element.

### 3.8. Pterygoid/quadrate overlap

We noted [1] a short overlap of the pterygoid and quadrate ([10], ch. 119; [5], ch. 123), a point queried by Brownstein *et al.* [5]. However, this is visible in the holotype and isolated quadrates in which the pterygoid facet on the lappet is restricted to the ventral medial surface (figure 2*d*,*e*) and not higher as in the rhynchocephalians, e.g. *†Clevosaurus* [12, figs. 13a, 17] where it occurs much more extensively in the middle and upper part of the anterior medial edge. It is true that the holotype quadrate has some damage medially, but the pterygoid lamella can be discerned in the scan, and separate quadrates from larger *†Cryptovaranoides* individuals are intact and demonstrate, from the pterygoid facet, that the overlap is indeed short. In fact, compared to sphenodontians, the quadrate has a short anterior development of the medial flange (figure 2*d*,*e*) so does not permit a long overlap. The posterior part of the holotype pterygoid is expanded into a small quadrate flange (figure 2*f*) that would have overlapped the quadrate. We consider that the evidence is robust in support of the claim of a short overlap [1].

### 3.9. Fusion of the premaxillae and single median tooth

*Contra* Brownstein *et al*. [5], we described [1] the premaxillae of *†Cryptovaranoides* as ‘premaxillae fused in *larger* individuals’ ([10], ch. 1; [5], ch. 1). We scored the character as {0,1} to include unfused premaxillae in juveniles and fused in adults, and so our neutral scoring cannot bias the results in favour of or against a squamate identity for *†Cryptovaranoides*. We also labelled an individual premaxilla of the holotype [1, fig. 1B]. A key feature of the *†Cryptovaranoides* premaxillae is that one bone (usually the right element) has four tooth emplacements whereas the left side has three, as in the holotype (figure 2*g*), an asymmetry we recorded [1, p. 9]. Here, we note that separate isolated premaxillae have three or four teeth, with all but one of the former on left bones and all the latter on the right bones. Premaxillae with four teeth are usually slightly larger (figure 2*g–i*). One premaxilla NHMUK PV R37378 [1, fig. 4B,C] is a partially fused bone at the base of both the anterior and posterior part of the suture line, but fused nonetheless. It is noticeably fused at the base when observed from the posterior side [1, fig. 4B] and in anterior view where the bone is fused with no suture just above the base of the median tooth [1, fig. 4C]. This specimen with fused premaxillae has four tooth emplacements on the right and three on the left, with the same tooth implantation as in the holotype.

The most medial of the right premaxillary tooth component of NHMUK PV R37378 is the median tooth. On this basis, the most medial tooth of the holotype right premaxilla is the most likely to develop into a median tooth and the position of this tooth (figure 2*g*) from the scan suggests that it is of a suitable form. Despite the comment by Brownstein *et al*. [5] that the specimen cannot be compared to the holotype as all teeth are broken (which they are at their crown apices), we are sure that NHMUK PV R37378 is a fused *†Cryptovaranoides* premaxilla. The same two posterior protuberances on NHMUK PV R37378 [1, fig. 4C] are also present on the separate isolated premaxillae shown in figure 2*h*,*i*. There are no other taxa with pleurodont premaxillae from the extensively researched fissures at Cromhall apart from the quite distinct rhynchocephalian *†Diphydontosaurus* with at least five teeth per element [14, fig. 5a]. No other taxon from British fissure deposits has the pleurodont premaxilla(e) composed of seven teeth, three on one premaxilla and four on the other. Note that we do not include the median tooth in the character list of Brownstein *et al*. [5] leaving it as written by those authors but do include it as an addition to the Tałanda *et al*. [10] matrix as the character applies to many of the extant squamates. We have also scored the premaxilla as unfused (ch. 1) using the Brownstein *et al*. dataset [6] and in the first of the two Tałanda *et al*. [10] analyses following an ultra-conservative approach, but we use unfused and fused in the last matrix (both ch. 1) as we are sure that NHMUK PV R37378 is from *†Cryptovaranoides*.

### 3.10. Peg-in-notch articulation with rod-shaped squamosal

We showed [1] that the squamosal and quadrate articulated at the posterior end of the squamosal ([10], ch. 123; [5], ch. 127), and Brownstein *et al*. [5] agree. We recognize that the squamosal lay in similar orientation to the postorbital and both are in their expected positions with respect to the right prefrontal. We observe the notch on the medial side of the cephalic head of the holotype quadrate (figure 2*d*,*e*) but also on several isolated bones, and a peg on the ventral surface of the posterior expansion of the squamosal that would fit that notch. There is no separate character for the squamosal peg in the latest data matrix [5], although in character 127 they include the squamosal contact with the quadrate notch.

### 3.11. Frontal underlaps parietal laterally on frontoparietal suture

Our observation [1] is that the frontal underlaps the parietal laterally [10, ch. 67−71] is countered by Brownstein *et al*. [5] who say that ‘the frontals are not preserved in the holotype and the referred frontals were found in isolation and cannot be anatomically connected to any of the other preserved elements in the skull without ambiguity’. It is correct that we stated [1] that there are no frontals in the holotype, and we assigned an isolated frontal NHMUK PV R37274 [1, fig. 7F–H] to *†Cryptovaranoides* based on the complementary morphology of the facets on the curved posterior process of the holotype prefrontal [1, figs. 1B, 6A] with those of the lateral facets of the frontal. The undoubted *†Cryptovaranoides* isolated prefrontals also have posterior process facets (figure 2*j*) that match those on the lateral side of the frontal. Furthermore, the ornamented surface of the prefrontal (figure 2*j*,*k*) matches that of the frontal. Importantly, we have recognized a small fragment of ornamented, shield-like bone, positioned in the left side skull orbit of the holotype (figure 2*b*) as deriving from a skull roofing bone, in the position below the frontal in life. We are therefore confident that our scoring for features of the frontal of *†Cryptovaranoides* is valid in the new data matrix [5, ch. 67−71]. As we can recognize the facet for a parietal in the position indicated before [1, fig. 7F–H) we confirm that it underlaps the parietal laterally. Note that the parietal facet underlapping is not included as a character in the character lists we use [5,10], and therefore makes no contribution to the phylogenetic analyses.

### 3.12. Articulars and prearticulars medial process

**Figure 3.**
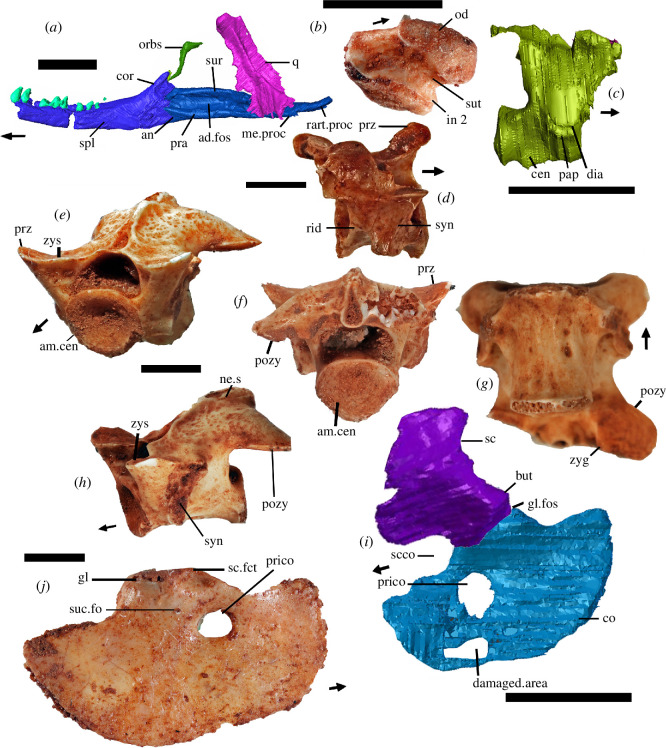
Holotype (*a,c,i*) and isolated bones of *†Cryptovaranoides microlanius*. (*a*) NHMUK PV R36822 CT scan of the right lower jaw, right quadrate and right orbitosphenoid in medial view of the lower jaw. (*b*) NHMUK PV 38897 isolated atlas centrum (=odontoid process) fused to intercentrum 2. (*c*) CV 3 from juvenile holotype from right side view but reversed for comparison with NHMUK PV R37276. (*d*) Isolated cervical vertebra NHMUK PV R37276, probably CV3 in right lateral view. (*e–h*) Isolated mid-dorsal vertebra NHMUK PV R37277 in (*e*) anterolateral, (*f*) posterior, (*g*) ventral and (*h*) lateral views. (*i*) Reconstruction of left scapulocoracoid from CT scan of NHMUK PV R36822 in lateral view. (*j*) Isolated right coracoid NHMUK PV R37960 in lateral view. All scale bars are 2 mm. Arrows show anterior. ad, adductor; am, amphicoelous; an, angular; but, buttress; cen, centrum; co, coracoid; cor, coronoid; dia, diapophysis; fct, facet; fo, foramen (foramina); fos, fossa; gl, glenoid; in, intercentrum; me, medial; ne, neural; od, odontoid process (=atlas centrum); orbs, orbitosphenoid; pap, parapophysis; pozy, posterior zygapophysis; pra, prearticular; prico, primary coracoid opening; proc, process; prz, prezygapophysis; psf, postfrontal; q, quadrate; r, right; rart, retroarticular; rid, ridge; s, spine; sc, scapula; scco, scapulocoracoid opening; spl, splenial; suc, supracoroacoid; sur, surangular; sut, suture; syn, synapophysis; zyg, zygantrum; zys, zygosphene.

We noted [1] a medial process on the articulars and prearticulars ([10], ch. 193; [5], ch. 195), a character rejected by Brownstein *et al*. [5]. We described it as a rudimentary process, but it is nevertheless present, *contra* [5], as shown in figure 3*a*. The process means that the mandible is about 10% thicker in ventral view at its most prominent position lying just posterior to and slightly below the medial cotyle. While not projecting so far ventrally as in most extant squamates (which is why we described the feature as rudimentary), the position of the medial process relative to the cotyles in the juvenile *†Cryptovaranoides* is otherwise like that in extant squamates where it is formed on the anterior region of the retroarticular process. We conclude that our observation is accurate but score the character as a query (?) because it is rudimentary, and so our scoring does not bias the phylogenetic analysis.

### 3.13. Bicapitate cervical ribs and cervical ribs with an anteriorly oriented process

We described [1] the cervical ribs as bicapitate ([10], ch. 259; [5], ch. 264) as we recognized that there were two points of articulation on the centrum (parapophysis) and the neural arch (diapophysis) and that the ribs are two-headed in descriptive terms as they have two separate projections. Describing them as single-headed would have biased the phylogenetic analysis in favour of a squamate affinity for *†Cryptovaranoides*, because modern squamate ribs are repeatedly described as unicapitate or single-headed. The bicapitate cervical ribs of *†Cryptovaranoides microlanius* (e.g. figure 1*e*) are little different from the rib of cervical vertebra 4 of *Sphenodon* [20, fig. 28] or the anterior cervical ribs of *Varanus* [21, fig. 1A] which also have two distinct articulations. It is worth noting then that some modern varanid lizards indeed show double-headed ribs [20, p. 252] contrary to the frequently reported statement that all ribs in (crown) squamates are single-headed [4]. Brownstein *et al*. [5] did not consider the lizard *Varanus* in their critique but preferred to suggest protorosaur or other archosauromorph affinities. However, in archosauromorphs the anterior process of the cervical ribs continues in line with the main part of the rib, forming an almost straight line, which is quite different from the feature observed in *†Cryptovaranoides*. Therefore, scoring this character as an archosauromorph feature [5] is inappropriate and biases their result. Note that the modern lizard *P. apodus* has an ‘anterior process’ on its fourth rib [22, fig. 9].

### 3.14. Cervical and dorsal vertebral intercentra

There are no dorsal intercentra in *†Cryptovaranoides* and we [1] scored the character ([10], ch. 237; [8], ch. 239; [5], ch. 239) as zero to document this, so we are puzzled why Brownstein *et al*. [5] headed their section ‘Cervical *and dorsal* vertebral intercentra present’. The lack of trunk intercentra, as in *†Cryptovaranoides*, is a squamate synapomorphy ([4,23]; although those authors consider that some geckoes and adult xantusiids have the character reversed).

Considering the cervical intercentra, we are sure that intercentra 1 and 2 are present (figure 1*e*) and intercentrum 2 is fused to the odontoid. In the matrix of Simões *et al*. [7], the cervical intercentra were not specified as postaxial intercentra, but these authors did reference [24] where the character is postaxial, and they are similarly not specified as postaxial in the text by Brownstein *et al*. [5]. We [1, fig. 1C, cer.in] considered a likely CV3 intercentrum, but following the discovery of a separate bone NHMUK PV 38897 (figure 3*b*), we now recognize the odontoid with fused intercentrum 2 (figure 1*e*), and another bone just posteriorly could be an intercentrum 3 but is perhaps more likely the axis centrum. Therefore, we have changed our scoring of this character from 1 to 0. Neither the odontoid/fused intercentrum 2 or the intercentrum 3/axis centrum are featured in the scans shown by Brownstein *et al*. [5].

### 3.15. Anterior dorsal vertebrae, diapophysis fuses to parapophysis

We observed [1] the separate diapophysis and parapophysis in the holotype CV3 (figure 3*c*) which fuse to form a synapophysis in a separate cervical vertebra PV R37276 (figure 3*c*). In the separate dorsal vertebra PV R37277 ([10], ch. 248; [5], ch. 252), the synapophyses are clear in the specimens (figure 3*e–h*). Brownstein *et al*. [5] state that, ‘even if synapophyses occur later in the ontogeny of *C. microlanius*, these are observed across several groups of reptiles, including all other non-squamate lepidosaurs and thus are not exclusive to squamates’. We do not doubt that and did not suggest that this was an apomorphy of Squamata [1]. We simply noted the feature in our description of *†Cryptovaranoides microlanius* and scored it as present in the dataset. An additional character [5, ch. 253] describes the shape of synapophyses on the posterior vertebrae, and we score this as query (?).

### 3.16. Zygosphene–zygantra in dorsal vertebrae

We observed rudimentary zygosphenes and zygantra ([10], ch. 246, 247; [5], ch. 249) in dorsal vertebra PV R37277, mentioned as a referred bone [1] and illustrated here (figure 3*e*,*g,h*). The presence of zygosphenes and zygantra is probably not diagnostic of Squamata, although they occur widely among squamates such as iguanids and snakes, but are also seen in non-squamate Lepidosauria, for example, in the basal rhynchocephalian *†Gephyrosaurus bridensis* [25]. Hoffstetter & Gasc [20, p. 236] define the zygosphene as rising medially from the prezygapophysis and the basic type is the ‘turning up of the prezygapophysial facet’ [20, p. 252] which is what we suggest is present in some isolated trunk vertebrae. In the case of *†Cryptovaranoides,* we can compare the small zygosphene articulation surface placed medially on the prezygophophysis (figure 3*e*) with those of the skink *Trachylepis quinquetaeniatam* as shown to demonstrate character 468 in [23, p. 237]. The feature is unobservable in the juvenile holotype and therefore to be conservative we do not score as present in the matrix of [10].

### 3.17. Anterior and posterior coracoid foramina/fenestra

We previously [1, fig. 7K] identified at least one foramen or fenestra in the coracoid but suggested that the more ventral of the two fenestrae ‘may be an artifact of the CT scan or a damaged area’, shown here also (figure 3*i*). However, the upper fenestra is real and not an example of ‘incomplete mineralization’, as suggested by Brownstein *et al*. [5]. In fact, the same fenestra is present in several separate *†Cryptovaranoides* coracoids in the collection (e.g. figure 3*j*). We regard it as the primary coracoid fenestra following the terminology of [11, figs. 1, 6]. As there, it is surrounded by either bone or (anteriorly) more likely calcified cartilage which is also the case in *Broadleysaurus major* [5, fig. 5a]. We did not code as present, ‘Character 284 procoracoid, coracoid emargination’ [8] as we cannot be sure that the procoracoid is emarginated. We thus score ([5], ch. 285; [10], ch. 282) as query (?) here. We did not describe any emargination of the coracoid or scapula [1], but rather there is an emargination between the scapula and coracoid (labelled ‘scco’ in figure 3*i*). This is the scapulocoracoid fenestra (=emargination) of Russell & Bauer [11] which we recorded from the holotype specimens that we figured [1, fig. S1G] and here (figure 3*i*).

## 4. Characters where absence or presence is equivocal

### 4.1. Atlas pleurocentrum fused to axis pleurocentrum

Brownstein *et al*. [5] state: ‘the intercentra (of the axis and atlas) are missing from the holotype’ and so cannot be assessed ([10], ch. 217; [5], ch. 220). That statement is certainly incorrect (figure 1*e*). Their statement that the atlas and axis pleurocentra are absent is also at least partly incorrect and possibly completely so. We [1, fig. 1C, ‘At.in’] had recognized the atlas intercentrum lying below the atlas neural arches in the holotype. We [1, fig. 1C] also labelled a bone as ‘cer.in’ which we now realize (following the discovery of an isolated bone NHMUK PV R38897, figure 3*b*) comprises the atlas centrum and intercentrum 2. The bone lying just posteriorly and next to the fused odontoid/intercentrum 2 could be the axis pleurocentrum but we need a better scan to be sure.; it may be intercentrum 3.

We have modified our scoring of the character ([5], ch. 220; [10], ch. 217] to 0 (absent) to indicate that there is no evidence of fusion of the atlas and axis pleurocentra. It may be that they are fused in larger individuals and the roughened posterior of the atlas centrum NHMUK PV R38897 suggests intimate contact, but we have no reliable evidence of fusion. We note that none of the elements, the atlas intercentrum, the fused odontoid/intercentrum 2 and the possible axis pleurocentrum appear on any of the scans in [5]. It is unclear why that is the case.

### 4.2. Presacral vertebrae midventral crest

A midventral keel ([10], ch. 229; [5], ch. 233, 234) is present on the pleurocentrum of cervical vertebra 3 but not connected to a hypophysis of the cervical vertebrae, and not a *crest*, which was the original character description [7, ch. 231] and remains the case [5, ch. 233, 234]. We [1, fig. 7N] figured an annotated cervical vertebra with a keel. Brownstein *et al*. [5] have reconfigured this character (without acknowledging the change) in their text to refer to either a *keel* or a crest. We have modified our scoring in the Brownstein *et al*. [5, ch. 233] matrix to meet their amended definition. Although Tałanda *et al*. [10, ch. 230] refer to a crest and not a keel, to be consistent we treat the character in the same definition as Brownstein *et al*. [5, ch. 233] and is therefore considered as present.

Brownstein *et al*. [5] state that we scored as absent ‘a midventral crest or keel on each caudal centrum’; that is untrue as we did not score a midventral crest or keel on any caudal centrum as none were present in the collection.

### 4.3. Angular does not extend posteriorly to reach articular condyle

Brownstein *et al*. [5] state that the ‘posterior portion of the angular is not observable in the holotype’ but indeed it is observable medially on the photograph of the right mandible [1, fig. 6D] and here (figure 3*a*) where it thins posteriorly to the mylohyoid foramen. Our interpretation is that the retroarticular process is composed of the articular which passes anteriorly to a unified surangular and prearticular element that starts as a suture line underlying the mandibular condyle. On the lateral side, there is a contoured feature that we interpreted as the posterior extent of the angular which underlies the mandibular condyle and which is the basis on which we reconstructed the mandible [1, fig. S1B,C]; there is no evidence of the angular behind the mandibular condyle to this position but a fused articular complex is present and therefore we retain our ‘informed interpretation’ [1, p. 11] on the nature of the posterior process of the angular. Note, however, that this character is not included in previous data matrices or the current ones [5,8,10] and therefore does not influence the phylogenetic analysis.

### 4.4. Ulnar patella

Brownstein *et al*. [5] state that we did not discuss the ulnar patella, but in fact we did [1, p. 12], where we reported that ‘there is no evidence of an epiphysis or a patella ulnaris in the ulna, perhaps because the holotype is a juvenile’. Such elements can readily be lost from fossil taxa [5], but these authors nevertheless concluded that its absence in the holotype of *†Cryptovaranoides* is likely to be real. We would not support that view.

**Figure 4.**
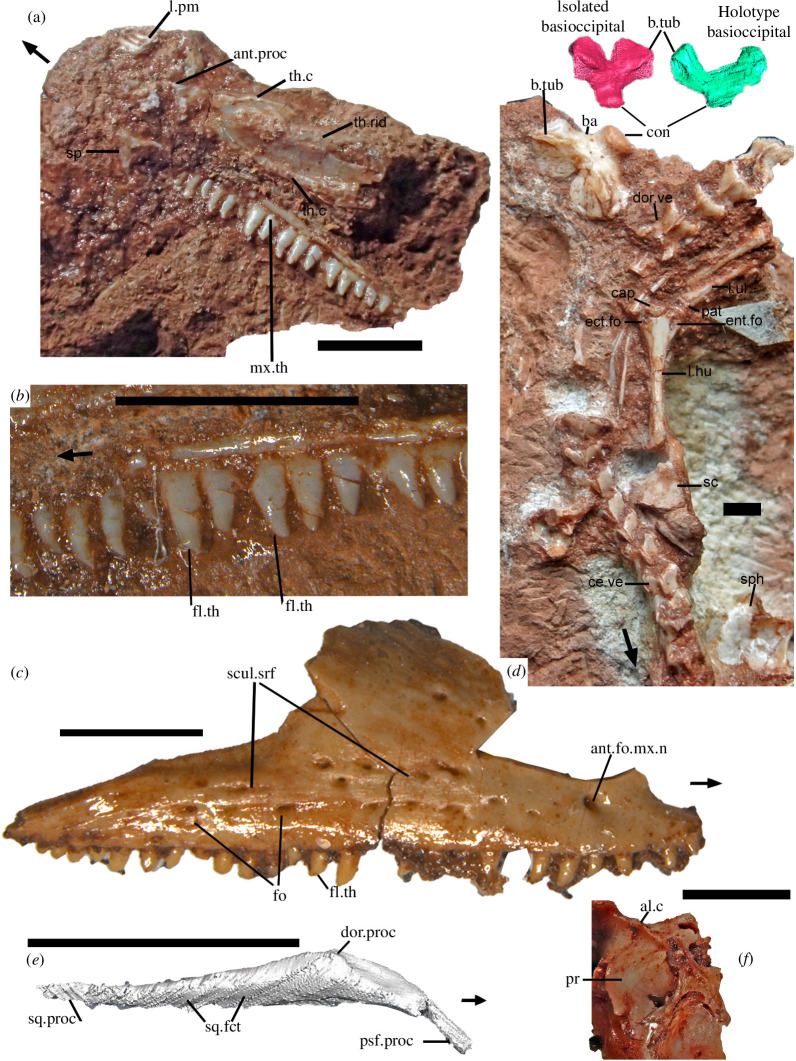
Cranial and postcranial bones of *†Cryptovaranoides microlanius*. (*a*) Close-up of *†Cryptovaranoides microlanius* holotype NHMUK PV R36822 right vomer and associated bones. (*b*) Detail of tooth row of right maxilla of NHMUK PV R36822 showing flanged teeth. (*c*) Right maxilla of *†Cryptovaranoides microlanius* (NHMUK PV R37279) to show distinctive multiple foramina and sculptured feature of the lateral surface and flanged tooth labelled. (*d*) Part of holotype rock NHMUK PV R36822 in the region of the cervical and dorsal vertebrae (mainly dorsal view) and including the isolated large basioccipital. The holotype basioccipital (in green) is shown at a similar size to the scan of the isolated basioccipital and in ventral view for comparison. The left humerus capitellum, entepicondylar and ectepicondylar foramina are labelled as is a dorsal vertebra in dorsal view for comparison with NHMUK PV R37277. The probable ulnar patella is also labelled. (*e*) Holotype right postorbital from CT scan in lateral view. (*f*) Section of prootic of isolated braincase NHMUK PV R R37377 showing alar crest. All scale bars are 2 mm but note that red and green scans of basioccipitals in (*c*) are not to scale. Arrows show anterior. al, alar; ant, anterior; b.tub, basal tubercle; ba, basioccipital; c, crest; cap, capitellum; ce, cervical; con, occipital condyle; dor. dorsal; ect, ectepicondylar; ent, entepicondylar; fct, facet; fl, flange(d); fo, foramen; l.hu, left humerus; l.pm, left premaxilla; l.ul. left ulna; mx.th, maxillary tooth; n, nerve; pat, patella; pr, prootic; proc, process; psf, postfrontal; sc, scapula; scul, sculptured; sp, septomaxilla; sph, sphenoid; sq, squamosal; srf, surface; th, tooth; th.c, toothed crest; th.rid, toothed ridge; v, vertebra; vo, vomer.

Therefore, we reconsidered the point and looked more closely at the holotype specimens rather than the scans which do not provide sufficient detail to decide if the ulna patella was absent. On examining the proximal head of the left ulna lying next to the distal end of the left humerus (figure 4*d*) we have concluded that the ulna patella is probably present in *†Cryptovaranoides* and is labelled in the figure. It shows the correct morphology, a thin discrete bone that caps the proximal end of the ulna and matches the lizard ulnar patella [11, fig. 1.1]. Having just discovered this bone we await further scanning to confirm or falsify our observations so for the present we code the character ‘?’.

## 5. Specific isolated bones of table 1 in Brownstein *et al*. (2023)

### 5.1. Toothed elements

*Contra* Brownstein *et al*. [5, table 1], we used the near-complete right dentary NHMUK PV R37281 to reconstruct the lower jaw [1, fig. S1C,D] and this is stated in the description of the figure. It was used in that reconstruction precisely because it was more gracile than NHMUK PV R37001 and had the greatest similarity to NHMUK PV R36822, the holotype right lower jaw. The pleurodont implantation and tooth form is the same on the holotype and NHMUK PV R37001. NHMUK PV R37281 has a split symphysis and is the same as that found in NHMUK PV R37001; both are certainly referable to the *†Cryptovaranoides* holotype dentaries.

The *†Cryptovaranoides* holotype left dentary has pronounced, consistently recurved pleurodont teeth (figure 2*b*), particularly in the mid-region, which is a unique feature among the extensively researched British Triassic fissure taxa. Of the other Cromhall fissure reptiles, only *†Diphydontosaurus* has pleurodont teeth (in mid and anterior dentary only) and, although some are recurved, that recurvature is substantially less pronounced and less consistent [14, figs. 28b, 29a,b] with a rounded crown apex rather than the sharp point found in *†Cryptovaranoides*. The carinae are difficult to spot on the holotype because it is heavily lacquered to protect the fossil bones, which is why we acquired the SEM image from an isolated specimen. However, the dentary fragment NHMUK PV R37282 is certainly referable to *†Cryptovaranoides* because of its strongly recurved shape, pointed crown apex and pleurodont implantation. The fossil assemblage from the *†Cryptovaranoides*-yielding matrix includes some *†Clevosaurus*, but only occasional *†Diphydontosaurus* and *†Kuehneosaurus* bones, all of which are well represented by bones in the NHMUK and elsewhere from Cromhall and other fissure deposits and are easily distinguished from *†Cryptovaranoides*.

In similar manner, the pleurodonty of the holotype maxilla (figures 1*c*, 2*b* and 4*a*,*b*) with relatively mesiodistally wide teeth (at the tooth base) in mid dentition including flanged forms (figure 4*b*), is uniquely characteristic of *†Cryptovaranoides* within the British fissure deposits and so the right maxilla NHMUK PV R37279 (figure 4*c*) is certainly referable to *†Cryptovaranoides*. NHMUK PV R37279 also has the same sculptured lateral surface and large foramina across the face (figure 4*c*) to NHMUK PV R36999 [1, fig. 3A], a left maxilla whose affinities to *†Cryptovaranoides* are not disputed by Brownstein *et al*. [5]. The flanged teeth of NHMUK PV R37280) shown on the SEM image [1, fig. 2F] are the same as the flanged maxillary teeth of the holotype (figure 4*b*) so it is also referable to *†Cryptovaranoides*.

### 5.2. Braincase

There is a basioccipital identified on the holotype scan [1, fig. 1C] lying below and posteriorly to the sphenoid (figure 1*e*). The broad lateral ‘wings’ in a fan shape (in dorsal or ventral view) and pronounced basal tubera are characteristic of *†Cryptovaranoides* and are found on the isolated basioccipital on the holotype rock (figure 4*d*; [1, fig. 1D]). Moreover, that basioccipital morphology is matched in the isolated braincase [1, fig. 4E]. So, the three specimens are united by this feature and are undoubtedly from the same taxon. The basioccipital is well-known in the rhynchocephalians *†Diphydontosaurus*, *†Gephyrosaurus* and *†Planocephalosaurus* ([14, fig. 26a,b], [19, figs. 36, 37], [26, pl. 71, fig. 3]) where the bone is distinctively sub-circular in dorsal or ventral view. In the reconstruction of *†Kuehneosaurus* by Robinson [27, fig. 1b] the trapezoid basioccipital is also quite different from *†Cryptovaranoides*. Therefore, no other lepidosauromorph or kuehneosaur taxa from British fissure deposits have the distinctive basioccipital found in the *†Cryptovaranoides* holotype. We also have a scan of the exoccipital part of the otoccipital from the holotype which has a vagus foramen (figure 1*g*,*h*). We have an isolated bone with the vagus foramen and two hypogossal foramina (figure 1*i*,*j*) and the latter unites NHMUK PV R38889 with the braincase NHMUK PV R37377 [1, fig. 4H]. Therefore, there are two lines of evidence demonstrate that the braincase NHMUK PV R37377 is from *†Cryptovaranoides* and we confirm it is indeed from that taxon.

### 5.3. Vertebrae

Brownstein *et al*. [5] contested the affinities of three vertebrae, cervical vertebra NHMUK PV R37276, dorsal vertebra NHMUK PV R37277 and sacral vertebra NHMUK PV R37275. While all three are amphicoelous and not notochordal, the first two can be directly compared to the holotype. Cervical vertebra NHMUK PV R37276 is of the same form as the holotype CV3 with matching neural spine, ventral keel (=crest) and the posterior lateral ridges or lamina (figure 3*c*,*d*) shown by Brownstein *et al*. [5, fig. 1a]. The difference is that NHMUK PV R37276 has a fused neural arch to the pleurocentrum and a synapophysis rather than separate diapophysis and parapophysis of the juvenile holotype (figure 3*c*). Neurocentral fusion of the neural arch and centrum can occur late in modern squamates, ‘up to 82% of the species maximum size’ [28].

The dorsal surface of dorsal vertebra NHMUK PV R37277 (figure 3*e*) can be matched to the mid-dorsal vertebra in the *†Cryptovaranoides* holotype (figure 4*d*, dor.ve) and has the same morphology of wide, dorsally and outwardly directed, prezygapophyses, downwardly directed postzygapophyses and similar neural spine. It is also of similar proportions to the holotype when viewed dorsally (figures 3*e* and 4*d*), both being about 1.2× anteroposteriorly longer than they are wide, measured across the posterior margin. The image in figure 4*d* demonstrates that the posterior vertebrae are part of the same spinal column as the truncated proximal region but the spinal column between the two parts is missing, probably lost in quarrying or fossil collection.

The sacral vertebra NHMUK PV R37275 is also amphicoelous but not notochordal and while we are sure it is from *†Cryptovaranoides* we agree that it cannot be matched directly to vertebrae in the holotype. Consequently, we have not used features from that specimen in our phylogenetic analyses. We will, however, revisit this specimen in a future publication.

The other fissure lepidosaurs, the rhynchocephalians such as *†Clevosaurus*, *†Planocephalosaurus* and *†Diphydontosaurus*, have notochordal amphicoelous vertebrae [12,26,29] so are not confused with the non-notochordal amphicoely of *†Cryptovaranoides*. Kueneosaurs have non-notochordal amphicoelous vertebrae, but the neural spine has a table at the apex [30, fig. 19D] on dorsal vertebrae and only one specimen of any *†Kuehneosaurus* bone, a quadrate, is recorded from the *†Cryptovaranoides* matrix.

### 5.4. Frontal

We have described our reasons for referring frontal NHMUK PV R37274 to *†Cryptovaranoides* above. In summary, we matched the posterior frontal facet on the holotype prefrontal and one on an isolated prefrontal (figure 2*j*) to the prefrontal facet on NHMUK PV R37274 [1, fig. 7F,G]. We now also recognize a fragment of frontal with the same shield structure in the holotype (figure 2*b*, labelled ‘f’).

### 5.5. Referring isolated bones to *†Cryptovaranoides*

The coronoid NHMUK PV R37273 is accepted by [5] as referable to *†Cryptovaranoides*. We emphasize that we always match isolated bones with their equivalents on the holotype and expand our explanations for the referral of isolated bones to *†Cryptovaranoides* in this section. The only bones that could not be directly matched with the holotype are the sacral vertebrae NHMUK PV R37275, but we are sure that they are from that taxon as they have the same characteristic amphicoely. We will return to those fused vertebrae in a later publication, but they have not been used to provide characters here.

We consider it remiss not to include isolated bones as they provide details which no scan can. Furthermore, as the holotype is a juvenile, they give additional information from larger, and presumably older individuals, on characters of the holotype otherwise unavailable or uncertain. There is a long tradition of study in the British Triassic and Jurassic fissures to reconstruct species based entirely on isolated bones such as *†Oligokyphus major* [31], *†Gephyrosaurus bridensis* [19,25], *†Planocephalosaurus robinsonae* [26,32], *†Diphydontosaurus avonis* [14] and *†Morganucodon watsoni* [33]. These animals were constructed from careful, meticulous observations of facets that matched one bone to another but from multiple individuals. Also, the taxon studied was invariably predominant in the deposit (90% of the faunal component, based on toothed elements, in the prepared rock in the case of *†Diphydontosaurus,* [14]). We note that Brownstein *et al*. [5] express concern about chimaeric organisms in the context of their discussions on the isolated bones of *†Cryptovaranoides* and expand the question to query more broadly the use of British Triassic fissure tetrapods in phylogenies. Yet Simões *et al*. [7] and Brownstein *et al*. [5] use published data from *†Gephyrosaurus*, *†Planocephalosaurus* and *†Diphydontosaurus* in their matrices with little reservation.

The difference in the case of *†Cryptovaranoides microlanius* is that we have an articulated holotype with which to compare isolated bones directly. We argue that our choice of bones [1] was entirely valid as we had in all cases, bar one, a direct comparison with a holotype specimen or in the case of the frontal, facets of a bone that matched. Furthermore, other taxa are very rare in the *†Cryptovaranoides* deposit, and their bones are recognizable from many papers over 70 years of research. In addition, we would assert that an analysis of isolated bones is essential to verify scan data and correct judgements made on scans that may be more poorly resolved than the researcher thinks. That is exemplified in this case as Brownstein *et al*. [5] did not recognize the entepicondylar or ectepicondylar foramina in the humerus, and yet they are manifestly clear in the actual holotype specimen as is the radial condyle (figure 4*d*). These authors seemingly also did not recognize the septomaxilla and the right jugal in the scans or the former on the specimen rock. Focusing on scan data alone or as the primary source can lead to major errors of interpretation and isolated bones can help to rectify the problem.

## 6. Phylogenetic analysis

We have compiled three taxon-character matrices using those of Brownstein *et al*. [5] and Tałanda *et al*. [10] as the basis for our phylogenetic analyses. We have scored the characters of *†Cryptovaranoides* using a conservative approach, based on our consideration of the evidence from the holotype fossil and isolated bones that are certainly referrable to the taxon, as explained in detail above. If we cannot provide substantiation that meets this standard, despite all evidence suggesting that the character is present (or absent) we code the character as ?.

We include some new bones identified from the holotype since our paper [1] and score for these on the matrices. The bones in the holotype are an orbitosphenoid (figures 1*e* and 3*a*), a bone identified as the postorbital [1, figs. 6D, 7C] but additionally, we now correctly recognize the postorbital (figure 4*e*) which is lying next to the squamosal. We shared an image of the former with Susan Evans and she (2023, personal communication) agrees that this is probably the orbitosphenoid. A calcified orbitosphenoid is a rare find in a Mesozoic fossil. The presence/absence of an orbitosphenoid is not a character in the new [5] matrix although its earlier use [10, ch. 158, 159] shows that it distinguishes many extant squamates. We also show the presence of a palpebral fossa on an isolated prefrontal (figure 2*k*) and an alar crest (figure 4*f*) on the braincase, as these contribute to the coding in both matrices. We further note that the ventral surface of the holotype vomer has three ridges or crests that converge anteriorly (figure 4*a*) including a relatively large longitudinal midline ridge (we therefore code 5, ch. 100; 10, ch. 92 as present) and demonstrate that it is not a flat bone and rather, is convex ventrally, so we code ([5], ch. 102;[10], ch. 95) as convex (1).

In that they have miscoded several key characters of *†Cryptovaranoides*, we cannot be confident that the phylogenies recovered by Brownstein *et al*. [5] are reliable. With minimal recoding to reflect the corrections we have described earlier, all our phylogenetic analyses, using diverse variants of the standard datasets [5,7–10] and analysed with parsimony or with Bayesian methods, always place *†Cryptovaranoides* within Pan-Squamata, sometimes in an unresolved polytomy with crown-clade Squamata, and when better resolved, within the crown Squamata as originally suggested [1]. Using Brownstein *et al*.’s [5] own data matrix demonstrates clearly that *†Cryptovaranoides* is not an archosauromorph.

**Figure 5.**
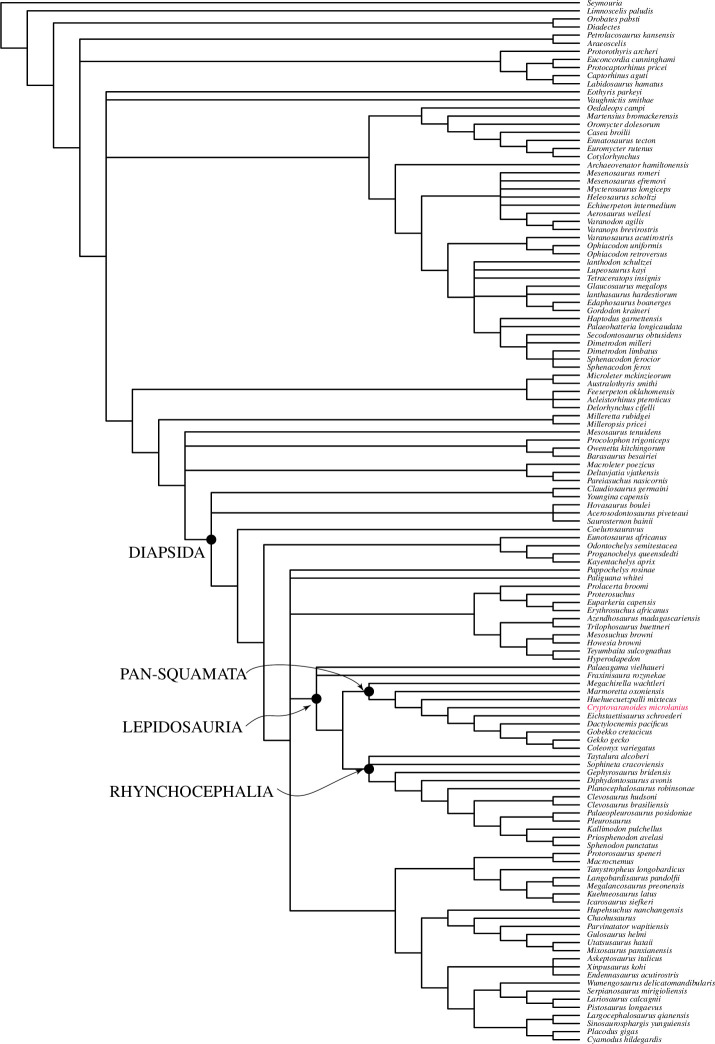
Bayesian phylogenetic tree processed in MrBayes based on the Brownstein *et al*. [5] dataset with characters of *†Cryptovaranoides microlanius* conservatively coded (see the electronic supplementary material for further details).

The character matrix used by Brownstein *et al*. [5] includes very few lizards, mainly just gekkotans and no unidentatans [34], so it is incapable of testing our original conclusion. The position of *†Cryptovaranoides* using this dataset is sister to the Gekkonomorpha and crownwards of *†Huehueuetzpalli* (figure 5). By contrast, the dataset of Tałanda *et al*. [10] includes over 50 living and extinct squamates and so enables a more focused phylogenetic analysis of the Lepidosauromorpha. The taxa sampled include a broad range of modern squamates, including geckoes, but also unidentatans, e.g. *Pseudopus* which uniquely in living lizards has vomerine teeth [18]. The Tałanda *et al*. [10] dataset is based on the modified Simões *et al*. [7] dataset with 347 characters for *†Megachirella* extensively modified by Griffiths *et al*. [35] researching *†Marmoretta oxoniensis*, who added over 30 new characters and finely checked all the scoring and reduced the number of taxa, followed on then by Ford *et al*. [36] on *†Paliguana whitei*. The Tałanda *et al*. [10] dataset is in turn modified from Ford *et al*. [36], thus providing a very detailed scrutiny of 382 characters including those that particularly differentiate squamates and stem squamates from other reptiles. Furthermore, Tałanda *et al*. [10] added additional taxa that are very close to the squamate clade, e.g. *†Bellairsia gracilis*, *†Oculudentavis naga* and *†Scandensia ciervensis*.

**Figure 6.**
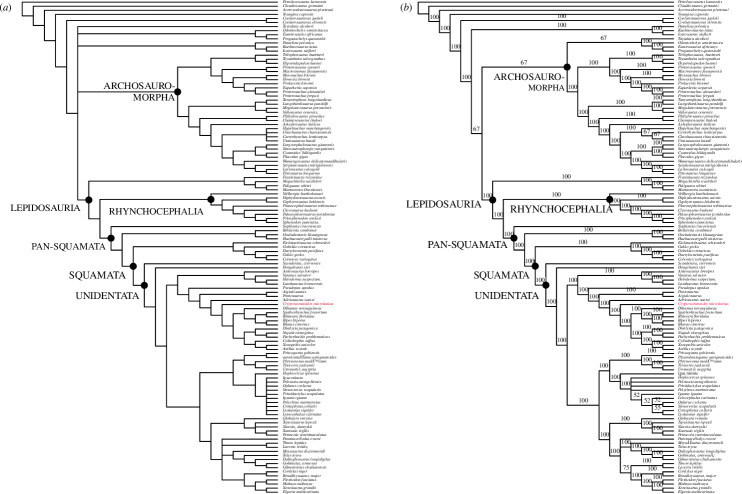
Parsimony phylogenetic trees based on the Tałanda *et al*. [10] dataset with characters of *†Cryptovaranoides microlanius* conservatively coded, including only unfused premaxillae for ch.1, and processed with PAUP. (*a*) Strict consensus and (*b*) majority-rule consensus.

*†Cryptovaranoides* is placed similarly in the majority-rule consensus tree (figure 6*b*) and is within a clade including the Anguimorpha, snakes and amphisbaenians, sister to a clade comprising the Iguania, Scincomorpha and Lacertoidea; *Elgaria* is again positioned anomalously. The Gekkonomorpha, *†Bellairsia*, *†Oculudentavis khaungraae* and *†Huehuecuetzpalli mixtecus* are again more basal. *†Cryptovaranoides* has a greater affinity to snakes than to the anguimorphs in both trees (figure 6*a*,*b*) than in our earlier study [1] but is found within a large clade that encompasses the Anguimorpha and Serpentes as well as the mosasaurs and amphisbaenians. Therefore, despite the highly conservative coding, *†Cryptovaranoides* is placed in a clade which includes the Anguimorpha as we found before [1] and is consistent with our earlier conclusion that *†Cryptovaranoides* is a Triassic crown-clade squamate and a unidentatan [34] within the crown Squamata.

Adding *†Cryptovaranoides* to the dataset of [10] in a morphological parsimony analysis places it in a strict consensus tree in a large clade within the crown-clade Squamata (figure 6*a*) that also includes most anguimorphs, snakes, mosasaurs, amphisbaenians, *†Ardeosaurus brevipes* [37] and dibamids. The Toxicofera [34] are split in this tree as a the Iguania are placed with other unidentatan squamates in a sister group including Scincomorpha, Lacertoidea and *†*Polyglyphanodontia with *Elgaria* as an anomaly (it is usually positioned as an anguimorph). The large clade of unidentatan squamates lies crownwards of *†Scandensia ciervensis* [38], *†Hongshanxi xeie* [39] and the Gekkonomorpha (including *†Eichstaettisaurus schroederi* [40]), while *†Bellairsia* [41], *†Oculudentavis khaungraae* [10] and *†Huehuecuetzpalli mixtecus* [42] are more basal. We treated the *†Cryptovaranoides* premaxillae as unfused in this analysis to encompass the juvenile holotype only.

**Figure 7.**
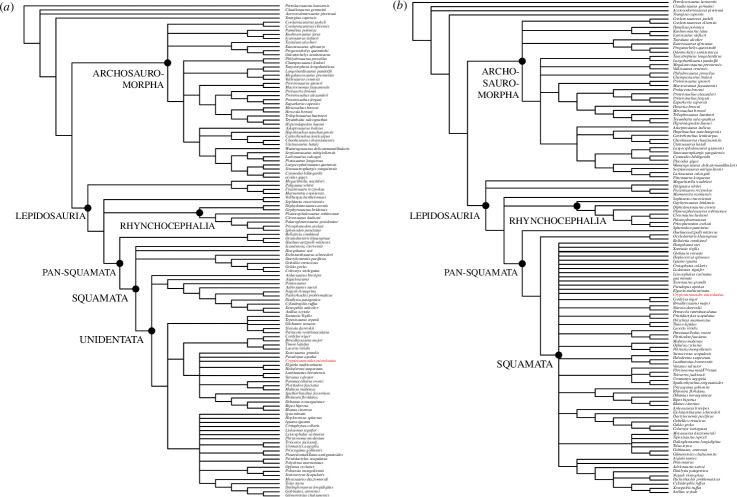
Bayesian phylogenetic trees based on the Tałanda *et al*. [10] dataset with characters of *†Cryptovaranoides microlanius* conservatively coded but with the addition of unfused/fused premaxillae and median tooth present/absent. (*a*) Without molecular constraints. (*b*) With molecular constraints and deletion of *Vellbergia* and *Scandensia* as rogue taxa, following [10].

When we considered the premaxillae as both fused (in adults) and unfused (in the holotype juvenile) and added character 383 that describes the presence of a median tooth in a Bayesian analysis (figure 7*a*) without the molecular constraints of [10] imposed, then *†Cryptovaranoides* is placed as a unidentatan [34] in a polytomy within the crown Squamata (figure 7*a*). It is in a clade with anguimorphs (including *Elgaria*), some scincomorphs, dibamids and amphisbaenians. The sister to this clade comprises some of the lacertoideans. These two clades are in a polytomy with two other groupings, one of which comprises Iguania, *†*Polyglyphanodontia [43] and *Teius*, a lacertoidean. The third clade in the polytomy includes *Xantusia, †Globaura* [44], *Petracola, †Slavoia* [45] and *†Tepexisaurus* [46]. The snakes and mosasaurs form a separate clade in the unidentatan polytomy which is completed by *†Ardeosaurus brevipes* [37] forming its own clade. The Gekkonomorpha, including *†Eichstaettisaurus schroederi* [40], lie more basally, in a polytomy with *†Hongshanxi xeie* [39] and *†Scandensia ciervensis* [38]. This grouping is at the base of the Squamata but is crownwards of the pan-squamates *†Oculudentavis khaungraae* [10], *†Bellairsia* [41] and *†Huehuequetzpalli mixtecus* [42].

When the same matrix is processed using the molecular constraints and methodology of [10], and removing *†Vellbergia* and *†Scandensia* as rogue taxa, *†Cryptovaranoides* is in a poorly resolved polytomy of unidentatan and gekkonomorph crown squamates (figure 7*b*). This includes *†Hongshanxi xiei* [39], *†Ardeosaurus brevipes* [37] and *†Eichstaettisaurus schroederi* [40] but all are crownward of *†Oculudentavis khaungraae* [10], *†Bellairsia* [41] and *†Huehuequetzpalli mixtecus* [42]. In these analyses based on morphology only and morphology with molecular constraints, *†Cryptovaranoides* is therefore certainly the oldest described crown squamate, as we have previously stated [1] and when better resolved is positioned as a unidentatan, crownwards of the Gekkonomorpha. It is far removed from the Archosauromorpha, *contra* Brownstein *et al*. [5].

## 7. Diagnostic characters of key clades

Following our phylogenetic analysis using the data matrix of [10], we can review the apomorphy distribution. *†Cryptovaranoides* shows three diagnostic characters of Lepidosauria given in [2], namely, lacrimal reduced or absent and largely confined to the orbital rim [1], teeth lost from transverse process of pterygoid and from sphenoid bones [1], marginal teeth attached superficially to lingual surface of jaw, rather than in shallow sockets [1]. Other ‘diagnostic apomorphies’ of Lepidosauria, defined in [2], cannot be determined because of missing fossil material (e.g. thyroid fenestra between pubis and ischium, fused astragalus and calcaneum, fusion of centrale to astralagus, loss of distal tarsals 1 and 5, metatarsal 5 hooked in two planes).

Furthermore, *†Cryptovaranoides* shares 10 apomorphies, all as defined by [3] and reported in [1], with the clade Pan-Squamata: (probably) angular does not extend posteriorly to reach articular condyle; coronoid eminence of the mandible formed by coronoid bone only, not in the combined process of the dentary and prominent dorsal expansion of the surangular; quadratojugal not present as a separate element; short overlap in quadrate-pterygoid contact; scapulocoracoid emargination/fenestration present; jugal posterior process absent; coronoid posteromedial process present; jugal closely approaches level of prefrontal below orbit; jugal entirely exposed above labial margin of maxilla; coronoid arches over dorsal face of mandible to reach lateral side of surangular.

*†Cryptovaranoides* has two diagnostic characters of Squamata (=crown-clade Squamata; we give the citation for the squamate synapomorphy at the end of each character), namely [1] the fused premaxillae (in adults) and the premaxillary median tooth [23]. Note, however, that the basal rhynchocephalian *†Gephyrosaurus bridensis* is scored (01) by [10] as some premaxillae specimens are fused. A further eight squamate synapomorphies are cephalic head of (mobile) quadrate with notch for the squamosal, peg-in-notch articulation with rod-shaped squamosal [4], vomer and maxilla meet at anterior margin of fenestra exochoanalis [23], prominent choanal fossa on anterior margin of ventral surface of palatine [23], subdivision of embryonic metotic fissure by the crista tuberalis into vagus (jugular) foramen and recessus scala tympani [4,7], no quadrate foramen [23], medially positioned posterior mylohyoidal foramen on mandible [23], fusion of exoccipitals and opisthotics forming an otoccipital [4,23], and trunk vertebrae lack intercentra [4].

We do not include the synapomorphy enclosed vidian canal exiting anteriorly at base of each basipterygoid process [23] in this analysis, as explained above. Three further synapomorphies of Squamata are not tested in the Tałanda *et al*. [10] matrix: palatine extends posteriorly so pterygoid enters suborbital fenestra [23]; coronoid anteromedial process fits into sulcus beneath tooth-bearing border of dentary [23] and frontal underlaps (or barely overlaps) parietal laterally on frontoparietal suture [23], but all three are present in *†Cryptovaranoides* [1]. An ulnar patella is probably present in *†Cryptovaranoides* but is not included in this analysis; it is a Pan-Squamata apomorphy as it is present in *†Megachirella* [7] rather than a synapomorphy of the Squamata as suggested by [5,47]. However, despite not including these synapomorphies nor the purported unidentatan or anguimorph apomorphies described below in the matrix of [10], *†Cryptovaranoides* is still placed as a (crown) squamate in the resulting trees.

Within Squamata, *†Cryptovaranoides* shows five synapomorphies of, or within, the Unidentata [1]: frontoparietal suture moderately interdigitated [23]; rugose ornamentation over dorsum [23], although some geckoes do have a rugosity over the dorsum [1]; jugal lies ventral to lacrimal [23]; posterodorsally trending ridge delineates anterior limits of nasolacrimal fossa [23]; and septomaxilla contacts dorsal surface of palatal shelf of maxilla [23], shown by presence of septomaxillary facet on *†Cryptovaranoides* maxilla [1]. However, as previously noted [1], *†Cryptovaranoides* has, plesiomorphically, amphicoelous rather than procoelous vertebrae found in all other unidentatans. Furthermore, *†Cryptovaranoides* shares four synapomorphies with Anguimorpha: frontal underlaps parietal laterally on frontoparietal suture (but also found in Iguania) [23]; short overlap or short lappet in quadrate-pterygoid contact [23]; long ventral longitudinal ridges converging towards midline of vomer [23]; and lacrimal arches dorsally over lacrimal duct and floors lacrimal duct with medial process posteriorly [23].

On the other hand, to identify *†Cryptovaranoides* as an archosauromorph requires the rejection of all these lepidosaur, pan-squamate and squamate characters, and the discovery of others to justify the alternative placement. Brownstein *et al*. [5] point to its possession of two traits: a strong anterior emargination of the maxillary nasal process (rare in squamates, common among archosauromorphs, where it contributes to the formation of the antorbital fenestra or fossa); and presence of an anterior process on the cervical ribs. We rejected both above. Their key reasons for doubting the lepidosauromorph affinities of *†Cryptovaranoides* relate to key traits of the distal end of the humerus, where these authors [5] emphasized the absence of three features, the ectepicondylar and entepicondylar foramina, radial condyle and ulnar patella. In fact, two of these features (ectepicondylar and entepicondylar foramina, radial condyle) are present. The possible absence of the ulnar patella was previously suggested by us [1] to reflect the young ontogenetic age of the *†Cryptovaranoides* specimen, as we or it may have simply been missed as it would be extremely small. We have now recognized the likely ulna patella directly from the holotype fossil (figure 4*d*) rather than in a scan.

More widely, if *†Cryptovaranoides* is an archosauromorph, we would expect it to show more than just two highly questionable diagnostic traits. Using information from recent phylogenetic analyses of Archelosauria and Archosauromorpha [47–50], we found very few diagnostic characters in the preserved elements. Archelosauria, the clade including Testudines plus Archosauromorpha, has been associated with differing synapomorphies. *†Cryptovaranoides* lacks three of the four diagnostic characters of Archelosauria in one recent analysis [47], absence of humeral entepicondylar foramen, laterosphenoids and lateral adductor crest on surangular. A fourth archelosaurian apomorphy is present however, a sagittal crest on the supraoccipital in the isolated braincase NHMUK PV R37377. Likewise, *†Cryptovaranoides* lacks the four synapomorphies of Archelosauria identified in another phylogenetic analysis [48]: frontal with distinct posterolateral processes; frontal anterior margins oblique, forming an angle of at least 30 degrees with long axis of the skull; interclavicle anterior process or triangle conspicuously present; and upper temporal fossae present and distinctly smaller than the orbit.

*†Cryptovaranoides* also lacks two of the three synapomorphies identified [49] as diagnostic of Archosauromorpha: posterior side of quadrate head expanded and hooked; and prominent tubercle developed superior to glenoid fossa of scapula. However, the lateral surface of the orbital margin of the frontal is rugose [1, fig. 7G]. The four synapomorphies diagnostic of Archosauromorpha identified in another phylogenetic analysis [50] are not observed in the present material of *†Cryptovaranoides*: at least one or more cervical or anterior dorsal vertebrae with a parallelogram centrum in lateral view [1, fig. C], in which the anterior articular surface is situated higher than the posterior one; posterior cervical and anterior dorsal vertebrae with anterior centrodiapophyseal or paradiapophyseal lamina [1, figs. 1C, 7N]; cervical and/or anterior dorsal vertebrae with posterior centrodiapophyseal lamina [1, figs. 1C, 7N]; posterior cervical and/or anterior dorsal vertebrae with prezygodiapophyseal lamina [1, figs. 1C, 7N]. It also lacks diagnostic traits of the more inclusive archosauromorph clades within which Brownstein *et al.* [5] place it within Crocopoda and sister to the Allokotosauria.

## 8. Conclusions

Brownstein *et al*. [5] state that they ‘robustly reject the crown squamate affinities of *†Cryptovaranoides*’ and repeatedly resolve the taxon as an archosauromorph. We question the robustness of their rejection of the squamate affinity of *†Cryptovaranoides* as it is based on critical omissions in their observations. Notably, Brownstein *et al*. [5] failed to recognize or to describe obvious features in the holotype such as the ectepicondylar and entepicondylar foramina and the radial condyle in the humerus, the presence of a septomaxilla and the presence of the right jugal. By scoring the humerus ectepicondylar and entepicondylar foramina as absent, despite the clear description and illustration of [1] earlier and emphasized in this paper. Their analysis is void because, as Brownstein *et al*. [5] state; ‘the absence of both foramina is considered a strong diagnostic trait for archosauromorphs, and it is one of the anatomical features supporting the sister group relationship between turtles and archosauromorphs’.

The pleurodont tooth implantation of *†Cryptovaranoides* is problematic for Brownstein *et al*. [5] because it is widely understood to be an indicator of squamate affinities [51]. They [5] interpret the strong pleurodonty of *†Cryptovaranoides* as somehow equivalent to the tooth implantation in non-lepidosauromorph taxa such as kuehneosaurs, which, rather, have a subthecodont dentition where the teeth are in shallow sockets (alveoli) in a groove on the alveolar margin [30, figs. 1A2, 4A3, 18C]. Furthermore, they [5] go on to speculate that pleurodonty might have been ‘a juvenile feature of thecodont neodiapsids’ because the teeth of embryonic alligators can appear to be pleurodont in that they are not initially attached to the jaw in sockets. However, this is a passing phase of very young embryos, and hardly relevant to the presumably hatched and older juvenile or subadult *†Cryptovaranoides*. Moreover, Bertin *et al*. [52] indicate that ‘teeth of most basal amniotes were implanted in shallow or deep sockets, so that the plesiomorphic state for dental implantation in amniotes was probably subthecodonty or thecodonty’, confirming the widely accepted evidence that pleurodonty is a derived squamate feature.

We have also found that Brownstein *et al*. [5] underestimated the relevance of the choanal fossa in the palatine, the anterior margin of which they regarded as ‘mediolaterally restricted’ and that its form was rare or absent in living lizards, despite published evidence to the contrary [15]. Furthermore, the suggestion that it is similar to a ‘choanal fossa’ in protorosaurs such as *†Tanystropheus* is not credible. Although there is an indentation on the anterior margin of the palatine in *†Tanystropheus hydroides*, it does not form a sulcus on the palatine ventral surface. In *†Tanystropheus longbardicus* there is a ‘fossa’ on the ventral surface of the palatine, but it is a channel (cf. figure 1*k*,*l*) rather than a squamate-like sulcus. Interestingly, although Brownstein *et al*. [5] made this a point of emphasis in the commonality they perceived between protorosaurs and *†Cryptovaranoides*, they were not sufficiently convinced to score this feature as present in *†Macrocnemus* and *†T. longobardicus;* rather they scored this feature as a query in their matrix.

Brownstein *et al*. [5] did not mention the large occipital recess in the braincase of *†Cryptovaranoides* as an important squamate feature, which is absent in *Sphenodon* and a likely adaptation in early Jurassic squamates [51]. Brownstein *et al*. [5] state that the lack of a posteroventral process of the jugal was possibly owing to a breakage in the left jugal but failed to consider that the right jugal was of similar morphology, and they seemingly ignored or overlooked the earlier description and illustrations [1]. Their suggestion of anterior emargination of the nasal process of the maxilla as a hint towards an antorbital fenestra, a feature of Archosauria, was incorrectly interpreted because the anterior part of the holotype left maxilla is missing (figure 2*b*) as the complete holotype right maxilla demonstrates.

The determination by Brownstein *et al*. [5] that *†Cryptovaranoides* was an archosauromorph led them to speculate that ‘It appears that *†C. microlanius* is part of a poorly known radiation of early small-bodied archosauromorphs, and highlights a potential new branch in the exceptional Triassic radiation of crown reptiles and demonstrates the probability that key small-bodied clades might still await discovery’. *†Cryptovaranoides* is not a ‘problematic fossil’ [5]; it is clearly a lepidosaur and a squamate lacking most key diagnostic archosauromorph apomorphies but with multiple lepidosaur, pan-squamate and squamate apomorphies. Paradoxically, the main character (perhaps the only character) that distinguishes *†Cryptovaranoides* from all other crown-clade squamates is the presence of the entepicondylar foramen on the humerus which is a plesiomorphic feature. It is worth noting also that the loss of both entepicondylar and ectepicondylar foramina occurred in a few extant squamate clades such as chameleons and teiids [11] but neither was included in the matrix of [5]. These lizards should probably be included in any analyses including archosauromorphs and lepidosauromorphs so that trees are not skewed by missing taxa with these characters.

We continue to develop our work on *†Cryptovaranoides microlanius* but no new information has countered the original conclusion of Whiteside *et al*. [1], that at the very least, it is a crown squamate and most likely a unidentatan with anguimorph affinities. We have provided explanations here that strengthen and enhance our original view and will continue to research *Cryptovaranoides* seeking evidence that further tests that view.

## Data Availability

All data needed to evaluate the conclusions in the paper are present in the Dryad Digital Repository [53]. All specimens referred to are deposited in the Natural History Museum, London (NHMUK). Supplementary material is available online [54].
